# Expanding the C-S-R framework to incorporate microbial interactions: evidence from methane-consuming communities

**DOI:** 10.3389/fmicb.2025.1589221

**Published:** 2025-05-12

**Authors:** Rui Wang, Yuru Chen, Binyan Zhai, Sascha M. B. Krause

**Affiliations:** School of Ecology and Environmental Sciences, East China Normal University, Shanghai, China

**Keywords:** methanotrophs, functional traits, ecophysiology, microbial communities, global change

## Abstract

Microbial interactions are critical in shaping community assembly and ecosystem functioning, yet classical ecological frameworks such as Grime’s Competitor-Stress Tolerator-Ruderal (C-S-R) model primarily emphasize individual traits, overlooking interspecies dependencies. Here, we propose an expansion of the C-S-R framework to incorporate microbial interactions, using methane-consuming communities in methane-fed microcosms as a model system. We present experimental data on both natural and synthetic methane-consuming communities derived from Lake Washington sediments, demonstrating that nitrate availability regulates community dynamics and life strategies. Under nitrogen limitation, the methanotroph *Methylomonas* adopts stress tolerance via nitrogen fixation but loses its competitive advantage under nitrate-rich conditions. These shifts are linked to the emergence of *Methylotenera*, a non-methanotrophic methylotroph that relies on cross-fed carbon from methanotrophs (e.g., *Methylobacter*) and alters competitive outcomes through metabolic coupling. Our findings show that survival strategies are shaped not only by intrinsic traits but also by interaction-based traits that redistribute resources and reshape ecological niches. By integrating these dynamics, we offer a novel perspective on the C-S-R framework that captures both individual and emergent behaviors, providing new insight into microbial community resilience and improving the predictive power of ecological models under environmental change.

## 1 Introduction

Understanding the relationships between biodiversity and ecosystem functioning (BEF) has been one of the most pressing scientific and social challenges for decades (Ali, 2023; Cardinale et al., 2012). These relationships have been studied by using richness and evenness in a classical taxonomic approach but resulted in taxonomic units that can still be enormously diverse both in functionality and ecology (Hoefman et al., 2014; Staley, 2006). In an effort towards a more predictive and quantitative science, microbial ecologists have focused on functional traits (Krause et al., 2014; Martiny et al., 2015; Romillac and Santorufo, 2021; Westoby et al., 2021). We refer to functional traits as any morphological or physiological characteristics that enable microorganisms to adapt to specific environments, including the genetic traits governing these adaptations (Krause et al., 2014).

The best-known ecological concept that incorporates the traits (in the broader sense) of organisms is the r- and K-selection framework (Dobzhansky, 1950), which has been adapted to describe microbial strategies. For example, the copiotroph-oligotroph continuum (representing microbes with rapid growth in resource-rich environments versus those adapted to resource-limited conditions) and spatial mobility (such as enzyme-derived mobility in resource-rich environments versus autotrophic mobility in environments with inorganic resources) serve as functional analogs to r-K selection. These concepts reflect the trade-offs between fast growth and reproduction (r-strategists) and resource efficiency and competition (K-strategists) in microbial populations (Allison and Vitousek, 2005; Fierer et al., 2007; Koch, 2001).

However, because these concepts typically fail to accommodate the vast diversity of microbial functions and life history strategies (Stone et al., 2023), the classical Competitor-Stress Tolerator-Ruderal (C-S-R) framework (Grime, 1974, 1977), originally developed for plants, was proposed as an alternative model for microbes (Ho et al., 2017; Ho et al., 2013). Unlike the linear r-K life strategy continuum, this framework provides a more accurate representation of microbial life strategies by integrating phylogenetic and functional data. It groups traits into community-level responses (primary life strategies), while also permitting mixed life strategies (secondary life strategies), thus offering greater flexibility to capture the extensive metabolic diversity of bacteria (Ho et al., 2013).

To better adapt the C-S-R framework to microbes, several alternative variations have been proposed over time. The earliest of these, the Y-A-S framework, was introduced by Malik and colleagues (Bittleston Leonora et al., 2021; Malik et al., 2020; Malik et al., 2019), categorizing microbial strategies into resource acquisition (Y), high-yield (A), and stress-tolerant (S) strategies. This framework emphasizes the trade-offs between extracellular enzyme production for resource degradation and growth efficiency, with important implications for carbon cycling and storage, as discussed by Ramin and Allison (2019) and Anthony et al. (2020). Quantifying Y-A-S traits often involves molecular techniques such as genome size analysis, ribosomal gene copy number, and functional gene expression (Kallenbach et al., 2016; Malik et al., 2018).

More recently, the C-S-O model was proposed by Wood et al. (2023), shifting the focus from disturbance to resource variability and foraging traits, offering a new perspective on microbial life strategies. These current adaptations, including the Y-A-S and C-S-O models, represent significant advancements in microbial functional ecology. However, they do not always capture the shifts in microbial behavior driven by interspecies interactions, especially in systems with cross-feeding of metabolites. For instance, some microbes may alter their gene expression and metabolism in response to the presence of another species, leading to the secretion of carbon and energy sources that support the growth of the cross-fed species (Krause et al., 2017).

In this perspective article, we discuss observed discrepancies between the performance (or “fitness”) of microbes at the individual level and their behavior or fitness in the presence of other species that interact in the environment. The existence of such discrepancies could have significant implications for the application of the C-S-R framework, requiring a re-evaluation of how to interpret interaction-driven trait shifts and how to integrate microbial interaction categories into the framework. To illustrate this point, we present a case study using methane-oxidizing bacteria (methanotrophs) as a model system. Methanotrophs play a crucial role in mitigating methane, a potent greenhouse gas, and have been extensively studied. They possess a well-characterized physiology, ecology, and diversity, along with a narrow substrate range, a large collection of representative isolates, and a clear link between function and identity (as discussed in Knief, 2015). Importantly, methanotrophs have already been used to assign life strategies according to the C-S-R framework (Ho et al., 2017; Ho et al., 2013), making them an ideal example for exploring these issues.

## 2 Methane-consuming communities in Lake Washington

Lake Washington, a freshwater ecosystem of significant ecological and biogeochemical importance, hosts methane-consuming microbial communities that exemplify the necessity of interspecies metabolic interdependence over individual species-centric dynamics (Chistoserdova, 2011). In this environment, stable isotope probing (SIP) experiments show that methane-derived carbon is primarily partitioned between the methanotroph genus *Methylobacter* and the non-methanotrophic methylotroph genus *Methylotenera*, even though other methanotrophs such as *Methylomonas* and *Methylosinus* are also present (Beck et al., 2013; Beck et al., 2015). These communities, dominated by *Methylobacter* and *Methylotenera*, form tightly coupled, interaction-driven functional clusters that drive methane consumption under low-oxygen, methane-rich conditions (Krause et al., 2017). Hence, the selective partnerships are not random but governed by metabolic compatibility.

The persistence of these partnerships in freshwater sediments challenges reductionist approaches that separate methanotrophs from their ecological environment. In particular, efforts to cultivate methanotrophs in pure cultures often fail to replicate their natural activities because they exclude auxiliary heterotrophs. Aerobic methane oxidation is increasingly recognized as a communal process, where methanotrophs and their partners form functional modules tailored to environmental gradients (Yu et al., 2019 and references therein). These interactions are not incidental but evolutionarily refined, enabling ecosystems to modulate methane fluxes dynamically.

### 2.1 Community dynamics in methane-fed microcosms

**Figure 1:**
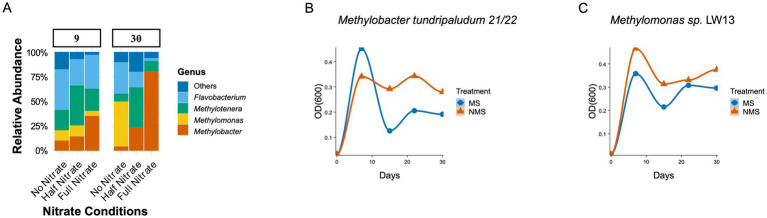
**(A)** Relative Abundance of microbial communities in different enrichments. Sediment slurries were prepared by mixing 6 grams of sediment from Lake Washington (LW) with 20 mL of nitrate mineral salt (NMS) medium in 100 mL vials (Dedysh and Dunfield, 2017). These vials were incubated under low-oxygen conditions with a gas mixture of 25% CH_4_, 1% O_2_, and 74% N_2_ to simulate O_2_ concentrations in the native LW sediment. The headspace gas mixture was renewed daily, resulting in recurring transitions between oxic and micro-oxic conditions, with weekly transfers and dilutions conducted as previously described (Hernandez et al., 2015; Oshkin et al., 2015). Three nitrogen treatments were applied: Full Nitrate (1 g/L KNO_3_), Half Nitrate (0.5 g/L KNO_3_), and No Nitrate (N_2_ as the sole nitrogen source). The microcosms were incubated for 30 days, with methane oxidation rates measured using gas chromatography. Changes in methanotrophic community structure were analyzed through Illumina sequencing of 16S rRNA at the 9th and 30th days. Sequences of 492 nucleotides length have been archived with the NCBI (Bioproject PRJNA310103). Illumina sequencing and the bioinformatics procedures have been described in detail in Hernandez et al. (2015). **(B,C)** Optical densities (OD600) were measured for pure cultures of *Methylobacter tundripaludum 21/22* and *Methylomonas sp.* LW13 in triplicates at 0, 7 15, 22, 30 days.

Lake Washington sediment was incubated in methane-fed microcosms under low-oxygen conditions (25% CH_4_, 1% O_2_, 74% N_2_) with three nitrogen treatments (see Figure 1). Under nitrogen-limited conditions (N_2_ as the sole nitrogen source), the genus *Methylomonas* dominated the methane-consuming community, with *Methylobacter* also present. These microbes, identified through 16S rRNA sequencing, are closely related to pure cultures isolated from the same environment, which both encode all the functions necessary for N_2_ fixation (Kalyuzhnaya et al., 2015). Our own pure culture studies showed that *Methylomonas* performs better than *Methylobacter* in N_2_-only treatments (Figures 1B,C). While differences in nitrogenase tolerance to O2 may play a role (Murrell and Dalton, 1983), these results align with the literature indicating variable nitrogen fixation in the genus *Methylomonas* but not in the genus *Methylobacter* (Auman et al., 2001; Semrau et al., 2011).

In contrast, the availability of nitrate shifts the community dynamics, favoring *Methylobacter* and *Methylotenera* as the dominant species (Figure 1; half and full nitrate). This finding aligns with previous studies in Lake Washington where these partnerships dominate the methane-consuming communities (Hernandez et al., 2015; Oshkin et al., 2015).

### 2.2 Synthetic microbial model communities demonstrate differential fitness

**Figure 2:**
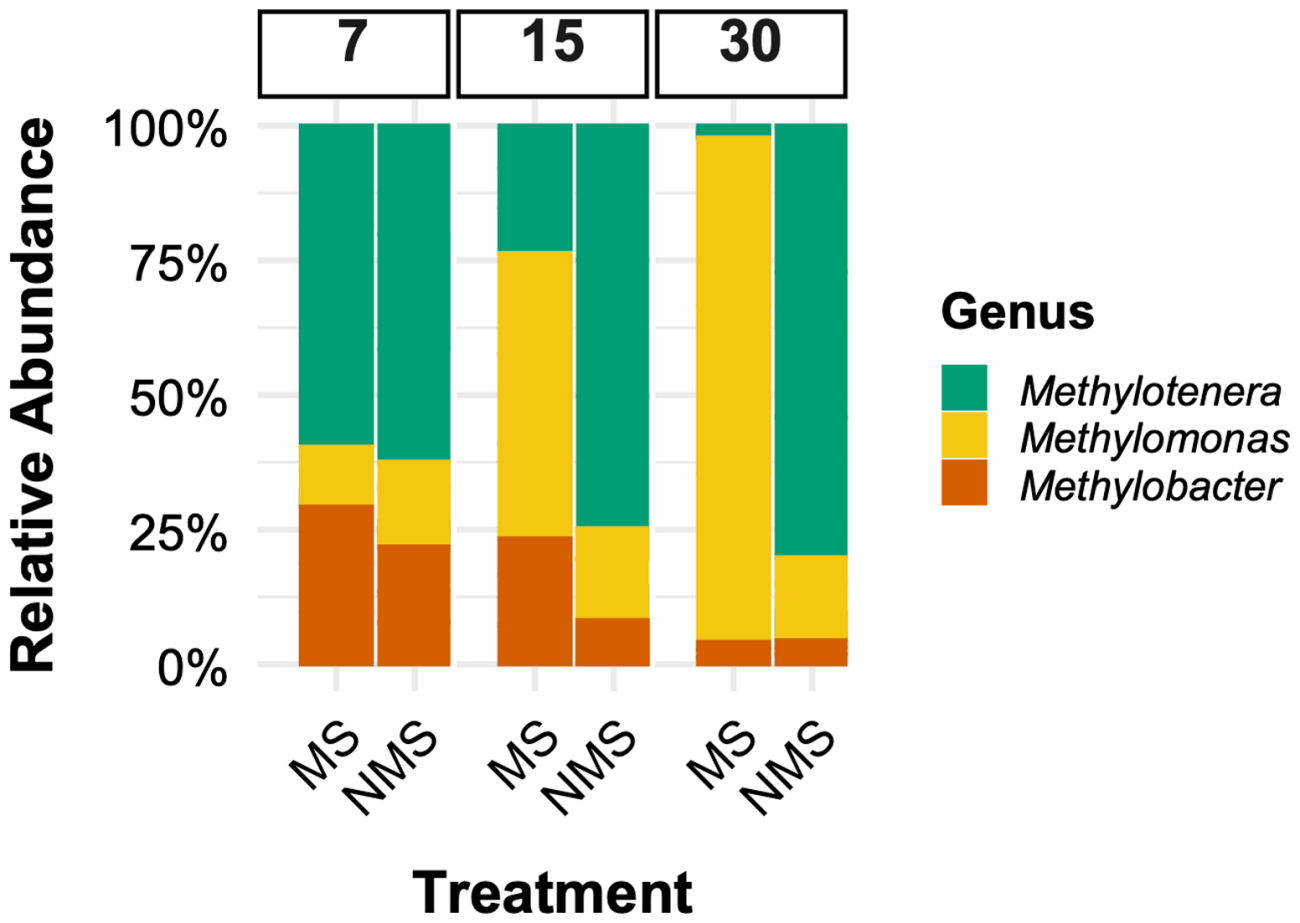
Relative abundance of microbial taxa (*Methylobacter tundripaludum 21/22*, *Methylomonas sp.* LW13, and *Methylotenera mobilis* 13) in synthetic communities. These are the representative strains isolated from Lake Washington (Beck et al., 2015) and were incubated under the same conditions as described in Figure 1. Each culture was initially inoculated at 2 × 10^8^ cells, as determined by flow cytometry (1,1,1 ratio). Two nitrogen conditions were tested [No nitrate (MS) and Full Nitrate (NMS), see Figure 1]. Despite using axenic cultures, high-throughput sequencing detected low-abundance taxa (≤1% of total reads). These unexpected sequences may result from intragenomic variation, PCR and sequencing errors, residual DNA in the culture media, index hopping artifacts, or transient contaminants introduced during handling. We did previously verify purity using selective media. To ensure our analyses focused on the core community dynamics, all minor taxa were excluded from statistical analyses. The 16S rRNA gene composition was analyzed on days 7, 15, and 30, following the protocol described by Liu et al. (2024). Sequences have been archived with the NCBI (Bioproject PRJNA1257058).

Synthetic communities composed of organisms involved in communal methane oxidation have been used as simplified, well-controlled models to validate findings from natural community experiments (Yu et al., 2019, and references therein). In methane-fed microcosms with NMS medium, synthetic two-species communities have shown that *Methylomonas sp.* LW13 consistently outcompetes *Methylobacter tundripaludum 21/22* (Yu et al., 2016, and references therein). We investigated the outcome of a similar experiment with a three-species methane-consuming community adding an auxiliary heterotroph: *Methylomonas sp.* LW13, *Methylobacter tundripaludum 21/22*, and *Methylotenera mobilis* 13, using the same incubation conditions (Figure 2). Our results show distinct competitive outcomes depending on nitrate availability. Under nitrate-limited conditions (N_2_ as the sole nitrogen source), *Methylomonas sp.* LW13 remained dominant (Figure 2), consistent with its competitive advantage observed in the sediment enrichments (Figure 1). However, under nitrate-rich conditions, *Methylomonas sp.* LW13 no longer dominated. Instead, *Methylotenera*, a methylotroph, became dominant (Figure 2), suggesting that *Methylotenera* alters community dynamics in nitrate-rich environments, through cross-feeding interactions (as demonstrated by Krause et al., 2017). *Methylobacter* remained the least abundant methanotroph under both conditions.

The persistence of *Methylomonas* in synthetic communities under nitrate-rich conditions (Figure 2), despite its exclusion from the natural enrichment (Figure 1A), likely results from the absence of ecological filtering by taxa or interactions not present in the simplified synthetic system. Similarly, the differing ratios of *Methylobacter* to *Methylotenera* between natural and synthetic communities underscore how context-dependent interactions, such as competition for electron acceptors and cross-fed substrates, shape community structure in ways that are not fully captured in the model system.

When assessing individual species’ responses within a functional framework like C-S-R, it is important to focus on how species behave in a community setting rather than in isolation. In our study, *Methylomonas* exhibits a notable shift in its competitive behavior when interacting with other species. While *Methylomonas* typically outcompetes *Methylobacter* in synthetic two-species communities, under nitrate supplementation in a community setting, *Methylomonas* loses its competitive advantage. *Methylotenera*, which cannot oxidize methane (Kalyuzhnaya et al., 2010), plays a supporting role by relying on byproducts released by *Methylomonas* and *Methylobacter* during methane oxidation. In the presence of nitrate in the medium, *Methylobacter* has been shown to provide metabolic by-products, such as methanol and formate, which can be utilized by *Methylotenera* (Krause et al., 2017; van Grinsven et al., 2021).

Furthermore, metabolic reconstruction of key species involved in methane consumption in Lake Washington has identified a potential link between methane oxidation and denitrification, indicating an adaptation to low-oxygen environments (Kalyuzhnaya et al., 2008). *Methylotenera* has denitrification genes (*narGHI*), allowing it to reduce nitrate to nitrite and possibly further, creating a micro-niche beneficial for both partners. This may help maintain redox balance and facilitate more efficient methane oxidation by *Methylobacter* under nitrate-rich, low-O₂ conditions (Liu et al., 2021). Likewise, the presence of nitrate has been shown to facilitate the transfer of methane-derived carbon from the methanotroph *Methylobacter* to the methylotroph *Methylotenera* in eutrophic lake water (van Grinsven et al., 2021). This interaction creates a feedback loop that shifts the community dynamics, suppressing *Methylomonas*’ typical dominance. These findings highlight the need to reconsider species’ roles within functional frameworks like C-S-R, accounting for the influence of interspecies interactions that can alter individual species’ behavior in community contexts.

## 3 Toward realistic models of methane-consuming bacterial communities: integrating nitrogen dynamics and interspecies interactions

Our experimental observations of *Methylomonas* and *Methylobacter* dominance under contrasting nitrogen regimes (Figure 1), alongside their partnership with *Methylotenera*, highlight the limitations of classical C-S-R theory in explaining microbial niche partitioning. Here, we reconcile these findings with thoughts on a revised C-S-R framework that accounts for metabolic interdependencies and cross-feeding interactions, drawing parallels to recent theoretical advancements in microbial trait ecology.

To clarify how microbial interactions reshape ecological strategies, we distinguish two categories of functional traits: individual traits—intrinsic characteristics encoded by an organism’s genome (e.g., nitrogen fixation in *Methylomonas*), and interaction-based traits—emergent properties arising from metabolic dependencies between species (e.g., *Methylobacter*’s reliance on *Methylotenera* for nitrate reduction). This distinction is critical for reconciling discrepancies between individual fitness and community-driven behavior, as illustrated in our case study. Below, we synthesize these insights into a revised framework that integrates metabolic interdependencies.

### 3.1 Competitor (C) strategies in resource-rich niches

*Methylobacter* exhibits traits consistent with a competitive strategy under nitrate-rich conditions (e.g., high-affinity nitrate uptake), but its ecological role in our system is shaped by more than intrinsic physiology (Figure 1). Its partnership with *Methylotenera* reflects interaction-based traits that support resource acquisition through mutual benefit: *Methylobacter* provides metabolic byproducts as a carbon source, while *Methylotenera* reduces nitrate to nitrite, potentially alleviating nitrogen competition (Li et al., 2024). This metabolic synergy exemplifies the “resource-use unconstrained” environments described in revised C-S-R models, where competitors amplify resource exploitation through cooperative interactions (Wood et al., 2023). These findings underscore the importance of interspecies networks in defining competitive outcomes and highlight a key limitation of classical C-S-R frameworks in capturing interaction-based traits.

### 3.2 Stress-tolerator (S) strategies in nitrogen-limited systems

*Methylomonas*’ nitrogen fixation is an individual trait, enabling survival in nitrogen-limited environments, stress tolerators prioritize survival over rapid growth, diverting resources to overcome environmental constraints (Wood et al., 2023). However, its suppression in nitrate-rich communities arises from interaction-based constraints (e.g., *Methylotenera*-mediated nitrate competition). Genomic evidence supports this: *Methylomonas* encodes nitrogenase complexes and alternative electron transport chains to sustain metabolism under oligotrophic conditions (Chowdhury and Dick, 2013). This aligns with revised C-S-R models where stress axes reflect “resource-use constraints,” such as the energetic cost of nitrogen fixation (Wood et al., 2023).

### 3.3 Revisiting ruderal strategies (R) in methane-oxidizing bacteria

Classical ruderal strategies emphasize post-disturbance recolonization, but methanotrophs redefine ruderality through nitrogen-phase adaptability. Under nitrogen-limited conditions, *Methylomonas* becomes the dominant species due to its ability to fix nitrogen through nitrogenase, which serves as an adaptation for stress tolerance. This aligns with revised C-S-R frameworks where “disturbance” encompasses metabolic instability, such as abrupt shifts in substrate supply. Although *Methylobacter* does not always dominate under nitrate-rich conditions, it exhibits traits consistent with a competitive strategy. Its persistence in the community is shaped by interaction-based traits, specifically its metabolic partnership with *Methylotenera*. This interaction-enabled ruderality, driven by interspecies cross-feeding, exemplifies a hybrid competitor-ruderal (C-R) strategy in which resource cycling replaces reliance on passive dispersal as the primary survival mechanism. This strategy combines individual stress tolerance, for example nitrogen fixation, with adaptability from interspecies interactions such as cross-feeding stabilization, thereby redefining ruderality as a hybrid trait.

### 3.4 Implications for microbial ecology and biogeochemical modeling

Our revised framework distinguishes individual traits (e.g., nitrogen fixation genes) from interaction-based traits (e.g., cross-feeding networks), resolving previous ambiguities in C-S-R classifications. This distinction is critical for predicting ecosystem responses, as interaction-based traits amplify or constrain individual strategies under environmental shifts. Incorporating interaction-driven strategies into C-S-R theory resolves longstanding discrepancies. For example, *Methylomonas* exhibits dual stress-tolerator/ruderal traits under nitrogen scarcity. *Methylobacter*’s hybrid C-R strategy emerges only in partnership with *Methylotenera*, highlighting context-dependent roles.

Future studies should prioritize quantifying metabolic costs of trait trade-offs: Measure metabolic costs of nitrogen fixation versus nitrate utilization (e.g., energy allocation in *Methylomonas* vs. carbon-sharing penalties in *Methylobacter*). To assess interspecies dependencies, we could apply stable isotope probing (SIP) to track carbon/nitrogen flux between *Methylobacter* and *Methylotenera* to quantify cross-fed metabolites (Radajewski et al., 2000) and metagenomics to profile functional gene abundances (e.g., *narGHI* for denitrification in *Methylotenera* and *nifH* for nitrogen fixation in *Methylomonas*) and predict community-level metabolic resilience (Pérez-Cobas et al., 2020).

## Data Availability

The original contributions presented in the study are publicly available. This data can be found here: https://www.ncbi.nlm.nih.gov/, accession number: PRJNA310103 and PRJNA1257058.
